# Exploding head syndrome's clinical features and an evaluation of polysomnographic findings

**DOI:** 10.1055/s-0046-1825765

**Published:** 2026-07-21

**Authors:** Kübra Mehel Metin, Selda Keskin Güler, Sinan Yetkin, Tahir Kurtuluş Yoldaş

**Affiliations:** 1 University of Health Sciences, Ankara Training and Research Hospital, Department of Neurology, Ankara, Turkey.; 2 University of Health Sciences, Gülhane Training and Research Hospital, Department of Psychiatry, Ankara, Turkey.; 3 Yıldırım Beyazıd University, Faculty of Medicine, Department of Neurology, Ankara, Turkey

**Keywords:** Parasomnias, Sleep, Polysomnography

## Abstract

**Background:**

Exploding head syndrome (EHS) is defined by the sudden onset of a short-term loud sounds or explosion-like sensation in the head during sleep, or sleep-wake transitions.

**Objective:**

To evaluate the clinical characteristics and polysomnographic (PSG) findings of patients diagnosed with EHS.

**Methods:**

The files of patients with an EHS diagnosis were retrospectively examined. Patients' clinical characteristics; PGS data; and results from the Epworth Sleepiness Scale (ESS), Pittsburgh Sleep Quality Index (PSQI) and Beck Depression and Anxiety Inventory were evaluated. Also, PSG findings were compared with control group data.

**Results:**

In total, 26 patients with EHS and 22 healthy controls were included in our study. The 16 patients who underwent PSG testing and the control group were similar in terms of age and gender. The total sleep time ( *p* = 0.001), sleep efficiency ( *p* < 0.001), and rapid eye movement (REM) percentage ( *p* = 0.019) were significantly lower in the patient group. The sleep latency ( *p* = 0.001), wakefulness after sleep onset ( *p* = 0.001), N1 percentage ( *p* = 0.010), and PSQI score ( *p* = 0.016) were significantly higher in the EHS group. Furthermore, 96% of the patients reported stress, and 84% of the patients had difficulties falling asleep.

**Conclusion:**

In this study the features of EHS are characterized and insomnia patterns emerge from PSG data. Examination with PSG should be considered in the presence of sleep disorders that disrupt sleep continuity. Patients' awareness about EHS should be increased, and people should be educated on taking sleep hygiene measures to strengthen sleep structure.

## INTRODUCTION

Exploding head syndrome (EHS) is a paroxysmal sensory parasomnia characterized by sudden loud sounds or an explosive sensation felt in the head during the transition from wakefulness to sleep or from sleep to wakefulness. 1 2 3 4 This condition is included in the other parasomnia group according to the International Classification of Sleep Disorders (ICSD-3). 5 Arousal and fear may follow the loud sound. This experience is not accompanied by a headache. 5

Previous research on polysomnography (PSG) or video electroencephalography (EEG) monitoring has demonstrated that episodes occur during the transition from wakefulness to sleep and from sleep to wakefulness. 6 7 8 However, due to its benign character and positive prognosis, EHS has not been the subject of extensive neurological research. 9 The PSG characteristics of EHS are still not completely reported and understood. Due to insufficient PSG data, it is necessary to clarify EHS with more detailed studies. 9 More research is necessary regarding sleep disruption, sleep fragmentation, and comorbid sleep disorders. Therefore, the aim of our study is to evaluate the clinical and PSG characteristics of patients diagnosed with EHS.

The term EHS was defined by Pearce in 1989. 1 Goadsby and Sharpless suggested that the definition of ‘exploding head syndrome’ was incomplete and that the term ‘episodic cranial sensory shock’, defined by Mitchell in 1876, should be used. 2 10 11 Gillis et al. reported a condition in which a sensory wave arises in the body and rushes to the head, as opposed to the common loud sound, and stated that this semiology supports the proposal to change the term EHS to ‘episodic cranial sensory shock’. 12

This syndrome is believed to be common but underreported. It is not well known by most clinicians. 1 13 14 The prevalence of EHS was found to be 11.1% 15 in a study with 180 participants and 18% 16 in a study with 211 undergraduate students. Among the Japanese population, the percentage who met the diagnostic criteria for EHS was 1.25%. 17

The differential diagnosis of EHS encompasses epilepsy or various types of headaches, such as hypnic, thunderclap, migraine, and cluster headaches. 18 There isn't extensive information about the etiopathogenesis of EHS. 19

## METHODS

Approval for our study (E-20/388) was received from the ethics committee of the Ankara Training and Research Hospital. The files of patients who were diagnosed with EHS and who had applied to the neurology outpatient clinic of the study's hospital, were retrospectively examined. The diagnosis was made through detailed clinical interviews according to the ICSD-3 criteria.

The patients applied to the outpatient clinic with complaints of hearing sudden and very loud sounds or feeling an explosive sensation while falling asleep or during sleep. Patients have stated that the episode they experienced was not a headache. During the episode, all patients experienced awakening and most were afraid. To exclude other diagnoses, patients underwent cranial magnetic resonance imaging (MRI), EEG and carotid vertebral doppler ultrasonography.

During the outpatient clinic visits, each participant was asked the same detailed questions about the characteristics of the sounds they heard, other accompanying conditions, and comorbid sleep conditions. For the clinical characteristics of the patients, PSG data and results from the Epworth sleepiness scale (ESS), Pittsburgh sleep quality index (PSQI), Beck depression inventory (BDI) and Beck anxiety inventory (BAI) were evaluated. The PSG findings were compared with data of age- and gender-matched healthy subjects without any medical, psychiatric, neurological or sleep disorders. Participants who were intellectually disabled or unable to answer the questions were not included in the study.

One night, PSG recordings were performed on patients with a Alice 6 PSG device (Philips N.V.). Electrooculography (EOG), chin electromyography (EMG), bilateral tibialis anterior EMG, EEG, and electrocardiography (ECG) were evaluated according to PSG American Academy of Sleep Disorders criteria. Patients' airflow, respiratory effort, oxygen saturation, and body position were recorded. The recordings were scored by the sleep physician according to the American Academy of Sleep Medicine (AASM) 2.3 criteria. 20 Sleepware G3 (Philips N.V.) software was used for PSG scoring. A respiratory event was scored as an apnea when both of the following criteria were met:

- There was a drop in the peak signal excursion by ≥ 90% of preevent baseline using an oronasal thermal sensor,
- Duration of the ≥ 90% drop in sensor signal was ≥ 10 seconds.

A respiratory event was scored as a hypopnea if each of the following criteria were met:

- Peak signal excursions dropped by ≥ 30% of preevent base line using nasal pressure;
- Duration of the ≥ 30% drop in signal excursion was ≥ 10 seconds;
- There was a ≥ 3% oxygen desaturation from preevent baseline, or the event was associated with an arousal.

The Apnea Hypopnea Index (AHI) was determined by counting the number of apneas and hypopneas per hour during sleep. Patients with an AHI value of ≥ 5/hour were determined to have sleep apneas. Insomnia was defined according to ICSD-3 criteria. 5

The PSQI is an 18-item questionnaire that measures sleep quality and disorders over a 1-month period. 21 A total score higher than 5 indicates someone who sleeps poorly. Furthermore, ESS measures participants' tendency to fall asleep in various situations; 22 the total score is the sum of eight ratings (range 0–24) with higher scores representing higher sleep. It has a cut off value of > 10 (abnormal condition) for daytime sleep. As for BDI, it is a 21-item questionnaire that evaluates the severity of depressive symptoms. 23 The standard cut off scores are as follows: 0 to 9 indicates minimal depression; 10 to 18 mild; 19 to 29 moderate; and 30 to 63 severe depression. Also, BAI is a 21-item questionnaire that evaluates the severity of anxiety. 24 The total score ranges from 0 to 63. Scores from 0 to 9 indicate normal or no anxiety; 10 to 18 indicate mild to moderate; 19 to 29 indicate moderate to severe, and 30 to 63 indicate severe.

### Statistical analysis

All analyses were performed on IBM SPSS Statistics for Windows (IBM Corp.) software, version 25.0. The Shapiro-Wilk test was used to determine whether continuous variables are normally distributed. Data are given as mean ± standard deviation (SD) or median (min-max) for continuous variables according to normality of distribution and as frequency (percentage) for categorical variables. Normally distributed continuous variables were analyzed with Student's test. Non-normally distributed continuous variables were analyzed with the Mann-Whitney U-test. Categorical variables were analyzed with the chi-squared test. Two-tailed *p* -values of less than 0.05 were considered statistically significant.

## RESULTS

The demographic data of the EHS patients and healthy controls were examined. We included 26 EHS patients (16 females, and 10 males), the mean age was 48 ± 15 (range 21–76). The PSG results were available for 16 patients (9 females and 7 males), mean age of these was 50 ± 13 (range 27–73). Also, 22 healthy controls (10 females, and 12 males) were included into the study, the mean age of these was 44 ± 7 (range 32–63). We found no significant differences between groups in terms of age ( *p* = 0.127) and sex ( *p* = 0.742). Furthermore, 14 (53%) of the patients were housewives and 10 (45%) were primary school graduates.

The clinical characteristics of EHS patients were evaluated. Duration of episodes was 1 to 2 seconds in 16 (61%) patients. Furthermore, 17 (65%) patients had episodes while falling asleep, 15 (57%) every day, and 9 (34%) heard the sound all over their head. Also, 11 (42%) patients had myoclonic jerks, 11 (42%) had tachycardia, and 22 (84%) showed fear ( Table 1 ).

**Table 1:** Summary of patient and disease characteristics

Patients and disease characteristics, N (%)		Total (n = 26)
**Duration of episodes, seconds**	1–2	16 (61.54%)
	3–5	4 (15.38%)
	5–10	2 (7.69%)
	10–60	4 (15.38%)
	60	0 (0.00%)
**Number of episodes per night**	1	12 (46.15%)
	1–3	4 (15.38%)
	3–5	7 (26.92%)
	5–10	1 (3.85%)
	10	2 (7.69%)
**Time of episodes**	While falling asleep	17 (65.38%)
	In the middle of sleep	4 (15.38%)
	While waking up from sleep	1 (3.85%)
	All of the above	0 (0.00%)
	While falling asleep and in the middle of sleep	3 (11.54%)
	While falling asleep and waking up from sleep	1 (3.85%)
	While waking up from sleep and in the middle of sleep	0 (0.00%)
**Duration of disease**	< 1 year	10 (38.46%)
	1–5 years	12 (46.15%)
	5–10 years	1 (3.85%)
	> 10 years	3 (11.54%)
**Frequency of episodes**	Every day	15 (57.69%)
	Once 2–3 days	4 (15.38%)
	Once a week	1 (3.85%)
	Once 2 weeks	2 (7.69%)
	Up to once a month	4 (15.38%)
**Type of noise**	Thunder	4 (15.38%)
	Ring tone	3 (11.54%)
	Glass breakage	2 (7.69%)
	Hitting	4 (15.38%)
	Hitting iron	2 (7.69%)
	Door slam	2 (7.69%)
	Explosion	3 (11.54%)
	Car engine	1 (3.85%)
	Piston	1 (3.85%)
	Hitting tin	2 (7.69%)
	Electric sizzle	1 (3.85%)
	Electrical switch knocking	1 (3.85%)
**Location of noise**	All over head	9 (34.62%)
	Both sides temporal	8 (30.77%)
	Both sides frontal	1 (3.85%)
	Both sides occipital	2 (7.69%)
	Left temporal	4 (15.38%)
	Right temporal	2 (7.69%)
**Myoclonia**		11 (42.31%)
**Tachycardia**		11 (42.31%)
**Fear**		22 (84.62%)
**Flash in the eye**		7 (26.92%)
**Stress**		25 (96.15%)
**Headache**		13 (50.00%)
**Dizziness**		7 (26.92%)
**Hypertension**		6 (23.08%)
**Other conditions**	Epilepsy	2 (7.69%)
	Hypothyroidism	4 (15.38%)
	Depression	1 (3.85%)
	Diabetes mellitus	2 (7.69%)
	Hearing problems	2 (7.69%)
	Tinnitus	2 (7.69%)
**Restless leg syndrome**		16 (61.54%)
**Hypnic jerk**		17 (65.38%)
**Snoring**		15 (57.69%)
**Difficulty in falling asleep**		22 (84.62%)
**Frequent waking**		17 (65.38%)
**Morning fatigue**		18 (69.23%)
**Daytime sleepiness**		12 (46.15%)
**Nightmare**		8 (30.77%)
**Sleep paralysis**		14 (53.85%)
**Sleep apnea**		8 (50.00%)
**Insomnia**		14 (53.85%)
**BDI score (mean ± SD)**		22.53 ± 10.99
**BAI score (mean ± SD)**		29.88 ± 16.42

Abbreviation: BAI, Beck anxiety inventory; BDI, Beck depression inventory; SD, standard deviation. Notes: Data are given as mean ± SD for continuous variables according to normality of distribution and as frequency (percentage) for categorical variables.

Comorbid conditions and sleep-related disorders in EHS patients were examined. In our cohort, 25 (96%) patients had stress, 13 (50%) headache, 16 (61%) restless leg syndrome, 22 (84%) difficulty in falling asleep, 17 (65%) frequent waking, 18 (69%) morning fatigue, and 14 (53%) had insomnia ( Table 1 ). The MRI results were normal 9 (50%), chronic ischemia 6 (33%), nonspecific hyperintensity 3 (16%).

**Figure 1:**
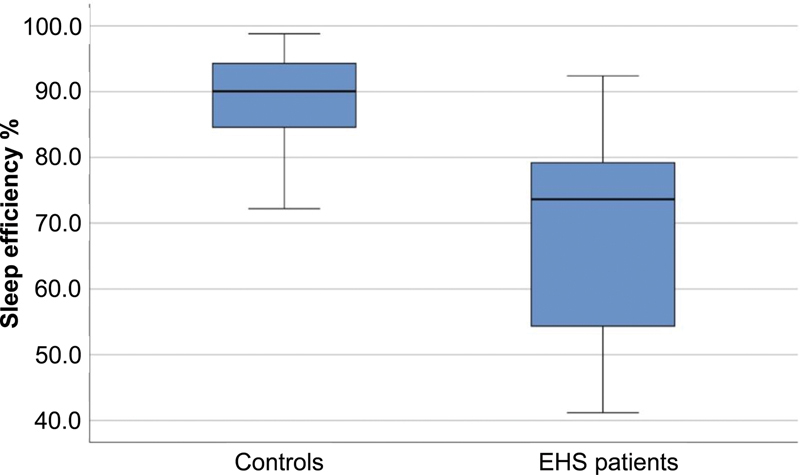
Sleep efficiency (%) with regard to controls and exploding head syndrome (EHS) patients ( *p* < 0.001).

The PSG findings were compared between EHS patients and control groups. Total sleep time ( *p* = 0.001), sleep efficiency ( *p* < 0.001) ( Figure 1 ) and rapid eye movement (REM) percentage ( *p* = 0.019) were significantly lower in patients than in controls. Sleep latency ( *p* = 0.001), wakefulness after sleep onset (WASO, *p* = 0.001), N1 percentage ( *p* = 0.010), apnea-hypopnea index (AHI, *p* = 0.017), periodic leg movement (PLM) index ( *p* = 0.045), time below oxygen saturation 90% ( *p* = 0.032) and PSQI score ( *p* = 0.016) were significantly higher in the patients group than in the controls group. Finally, 8 (50%) patients had sleep apnea. We found no significant differences between groups in terms of arousal index, PLM arousal index, desaturation index and ESS score ( Table 2 ).

**Table 2:** Summary of demographics and polysomnography results with regard to groups

		Groups		
		Controls (n = 22)	Patients (n = 16)	*p* -value
**Age (Mean ± SD)**		44.59 ± 7.87	50.50 ± 13.32	0.127
**Sex**	Female	10 (45.45%)	9 (56.25%)	0.742
	Male	12 (54.55%)	7 (43.75%)	
** BMI, kg/m ^2^**		26.25 ± 4.63	27.75 ± 3.57	0.285
**Total record time, min (mean ± SD)**		475.98 ± 52.93	487.04 ± 54.33	0.533
**Total sleep time, min (mean ± SD)**		424.95 (225.5–508.2)	334.9 (214.5–460.8)	**0.001**
**Sleep latency, min**		9.25 (1.0–26.5)	21.75 (3.0–94.0)	**0.001**
**Sleep efficiency, %**		90.05 (72.20–98.80)	73.65 (41.20–92.40)	**< 0.001**
**WASO, min**		36.77 ± 21.02	120.67 ± 77.92	**0.001**
**N1, %**		15.75 (6.4–26.9)	21.3 (8.2–72.5)	**0.010**
**N2, %**		50.27 ± 9.68	45.34 ± 16.14	0.248
**N3, %**		16.25 ± 7.04	15.33 ± 10.72	0.749
**REM, %**		18.05 ± 5.06	13.37 ± 6.69	**0.019**
**AHI**		2.6 (0.2–5.0)	4.7 (0.0–117.8)	**0.017**
**REM-AHI**		4.95 (0.0–21.1)	12.3 (0.0–80.5)	**0.010**
**PLM index**		1.35 (0.0–21.7)	8.85 (0.0–57.3)	**0.045**
**PLM arousal index**		1.25 (0.67–2.23)	2.0 (0.7–4.6)	0.204
**Arousal index**		3.35 (1.1–24.4)	6.0 (0.8–32.6)	0.080
**Desaturation index**		2.45 (0.2–6.2)	3.35 (0.0–128.9)	0.089
**Time below 90%, min**		0.1 (0.0–1.2)	0.4 (0.0–121.7)	**0.032**
**Minimum oxygen saturation**		91 (85–95)	89.5 (72–93)	0.220
**Average oxygen saturation**		95 (92–99)	94.5 (91–96)	0.457
**ESS score**		9.12 ± 5.99	6.33 ± 3.77	0.123
**PSQI score**		7.43 ± 4.47	12.20 ± 5.44	**0.016**

Abbreviation: AHI, apnea-hypopnea index; BMI, body mass index; ESS, Epworth sleepiness scale; PLM, periodic leg movement; PSQI, Pittsburgh sleep quality index; REM, rapid eye movement; SD, standard deviation; WASO, wakefulness after sleep onset.

Notes: Data are presented as mean ± SD or median (min-max) for continuous variables according to normality of distribution and as frequency (percentage) for categorical variables.

## DISCUSSION

In the current study, the clinical and PSG characteristics of EHS patients were evaluated in detail. In 65% of EHS patients, the episodes occurred while falling asleep. Also, 84% of the patients had difficulties falling asleep, and 96% of the EHS patients reported stress. The total sleep time, sleep efficiency, and REM percentage were significantly lower in the EHS group. The sleep latency, WASO, N1 percentage, and PSQI score were significantly higher in the EHS group.

Although EHS has been reported at all ages, it is predominantly observed in older people, and the average age of onset is 54 years 3 . The female-male ratio is 1.5 to 1. 3 4 One study found that EHS is quite common in young individuals and is not more common in women. 16 In our study, the female-male ratio was found to be 1.6 to 1, similar to the literature, and the average age was 48.

One publication stated that in half of the cases, the episodes have a highly variable frequency, ranging from one attack every few days to more than one per night, and that a chronic course is generally observed, while the episodic course is less frequent. 3 These sounds start suddenly and are typically brief in duration. 4 In our study, the syndrome was longer (under 5 years), and the rate of those experiencing daily episodes was 57%. In our study, in more than half of the cases, the episodes lasted 1 to 2 seconds and mostly occurred once per night. One publication stated that sounds were heard in the head and in both ears. 25 26 In another publication, sounds were felt on the right side in 26% of cases, on the left side in 19% and bilaterally in 55%. 25 In our study, during the attack, the sound was felt all over the head and bilaterally temporally.

In the case studies of Frese et al., light flashes were observed in 22% of patients, and fear was a prominent feature of the attacks. 3 In another publication, the rate of light flashes was 26.3%, and fear, tachycardia, muscle twitching, and muscle jerking were observed in most of the episodes. 25 Vision problems may be present in 10% of cases, along with fear, shock, confusion, tachycardia, palpitation, epigastric and precordial sensations, and electrical sensations rising from the lower part of the body to the head. 1 4 27 28 29 In our study, the light flashes were observed at a rate of 26%, a rate similar to that found in the literature. Additionally, in our study, fear accompanied loud sounds in 84% of cases. The rate of myoclonus and tachycardia was also high. In Sharpless's review of EHS, 27 different sounds were mentioned. 4 Our study registered 12.

One publication stated that 65% of the episodes occurred during the transition phase. 3 Another publication stated that EHS may occur during REM. 21 In 65% of our cases, the episodes occurred while the patient was falling asleep.

In the Sachs and Svanborg case series, no abnormal brain activity was observed in the 24 EHS episodes regarding PSG. 27 In the literature, no epileptic activity was observed in EEG during EHS episodes. 6 12 30 Since deep electrode recordings show subcortical seizure activity without cortical activity, the absence of epileptiform activity in the cortical EEG may not eliminate the possible epileptic basis of EHS. 4 During episodes, EEGs show that dominant alpha activity is with interspersed theta activity. 26 27 Furthermore, a warning effect in the form of a sudden increase in electromyogram (EMG) and beta activity and a simultaneous alpha blockade may be observed before EHS episodes. 26 27 Limited PSG examinations have not been able to establish a clear relationship with specific sleep stages. 6 7 8 27

In a retrospective case-control study, interictal EEG biomarkers were investigated by performing macrostructural and event-related dynamic spectral analyses of all-night scans. Additional oscillatory activity was observed during wakefulness and sleep/wake periods in patients with EHS. This activity was found to be different from the alpha rhythm it accompanies in terms of its frequency, topography, and source. 2 In our study, in the patient diagnosed with epilepsy, an EHS attack was observed in the PSG recording while he was transitioning from wakefulness to sleep (in phase N1), and no epileptic activity was observed at that time.

In the literature, hypertension, headache, diabetes mellitus, glaucoma, dizziness, sleep apnea, and restless legs syndrome are identified as comorbid diseases. 1 3 None of the cases in our study were characterized by EHS attacks accompanied by headaches, but half of them had comorbid headaches. A case with EHS followed by sleep paralysis and a migraine-type headache was reported, being observed as a migraine aura. 31 A relationship between sleep paralysis and EHS has been reported. 32 It has been stated that the high frequency of comorbid sleep disorders, especially isolated sleep paralysis, in EHS may suggest a common mechanism. 15 In our study, other accompanying diseases were dizziness, hypertension, hypothyroidism, hearing problems, epilepsy, tinnitus, diabetes mellitus, and depression. The most common sleep-related complaint was difficulty falling asleep, which may be explained by more frequent episodes happening at such times. In our study, sleep apnea was detected in 50% of the patients who underwent PSG. In some cases in the literature, sleep apnea was also detected. 9

There is a potential relation between EHS and emotional stress, as most patients report stressful life situations during periods when attacks are intense and frequent. 9 Feketeova et al.'s case, stress and anxiety can trigger attacks at night. 30 In our study, the presence of a stress factor in 96% of the cases and the high scores of the BDI and BAI suggested that the episodes may be related to such factors. The EHS attacks often cause fear and distress in patients. 4 13 Kaneko et al. reported a case involving recurrent panic attacks due to EHS attacks. 13 In one of our cases, EHS attacks started to occur while the patient was falling asleep due to intense anxiety, which is known to trigger EHS episodes, as in our case. Alternatively, fear caused by EHS episodes may trigger a panic attack, as in the literature. 13

When EHS attacks recur frequently, they may cause arousal and insomnia. 9 Denis et al.'s study found that insomnia symptoms independently predicted the presence of EHS, and the condition was associated with a variety of abnormal sleep experiences. 14 Sharpless et al. reported a cross-sectional study that people with EHS had shorter sleep times, longer times to fall asleep, lower sleep quality, and lower sleep efficiency, but effect sizes for these differences were small. 33

In the current study, the total sleep time, sleep efficiency, and stage percentage were significantly lower in the patient group than in the controls, while the sleep latency, WASO, and the N1 stage percentage were found to be significantly higher in PSG, indicating a deterioration in sleep maintenance and structure in EHS patients. Furthermore, we found that the PSQI was significantly higher in the patient group than in the control group. Additionally, any underlying sleep problem may have disrupted the sleep maintenance and structure and caused EHS attacks. In the case report of Nakayama et al., WASO was found to be 155 minutes; sleep efficiency was low; and sleep latency was long in the PSG of an EHS patient with restless legs syndrome. 9 Normal sleep structure was observed in two cases in the literature. 6 30 The PSG results showed that sleep continuity and structure were impaired in patients with EHS due to recurrent attacks. Patients who experience chronic frequent episodes may be prone to developing insomnia.

In the literature, the tricyclic antidepressants clomipramine, 27 amitriptyline, 19 calcium channel blockers flunarizine 34 and slow-release nifedipine, 35 anticonvulsant topiramate, 33 carbamazepine, 22 and clobazam 15 have been recommended in the literature for the treatment of EHS. Puledda et al. stated that transcranial magnetic stimulation (sTMS) could be a treatment option for EHS patients. 36 Generally, education, sleep hygiene, reassurance, and treatment of accompanying sleep disruptions and stress, result in improvements. 4 12 19 27 29

In the majority of cases, firm reassurance about the benign nature of EHS may be advisable, and drug therapy may not be necessary. 3 It has been demonstrated that attacks can be treated by treating other sleep disorders or concurrent diseases, such as depression, as the first treatment method for EHS. 9 According to Okura et al., 8 in a patient with accompanying obstructive sleep apnea, the EHS attack regressed when they started using an oral appliance. Stress and anxiety levels can perhaps be reduced through various relaxation techniques, meditation, or hypnosis. 4 It is important to note that 8% of those with recurrent EHS attempted to prevent attacks. 25

Prognoses are variable, 4 27 and insomnia resulting from EHS episodes may impair quality of life. 3 In our study, the attacks of some patients regressed spontaneously. However, amitriptyline caused attacks in one patient. Treatment for sleep disorders and comorbid psychiatric illnesses was recommended. It was observed that the patients were relieved after being diagnosed and learning of the benign characteristics of the disease. This confirms that it is important to provide sleep hygiene education and reassurance, as stated in the literature.

There are several limitations to our study. The small number of patients in our patient group was a limitation of our study. The patients answered the question about the sounds' duration according to their own perception. When the patients had an episode, they did not make a quantitative measurement of the duration. The patients who said it was longer than 10 seconds were indicating that the event they experienced was sudden. Since patients say the sound was sudden, it is possible the duration they stated may not be the actual duration and, instead, may be shorter. Another limitation of study, sleep efficiency and sleep latency are not based on 1 to 2 weeks of diaries, based on the data gathered on the night of their PSG. Monitoring patients with a sleep diary could also provide information about sleep characteristics.

In conclusion, we believe that our study will contribute to the literature by serving as a case-controlled study in which PSG data and the clinical characteristics of EHS patients are evaluated in detail. This case series further characterizes features of EHS and that an insomnia pattern emerges from PSG data. These findings are nonspecific and consistent with an insomnia pattern. These are changes in sleep that can be seen in association with stress and anxiety, as well as hyperarousal. In the case of EHS, the condition may cause disruptions in sleep continuity and structure, and vice versa. Therefore, evaluating, monitoring, and planning treatments for patients with PSG is important to detect comorbid sleep disorders. Additionally, our study confirmed that stress is an important factor in the occurrence of EHS attacks. Having a diagnosis with benign features will provide relief to patients. Therefore, disease awareness should increase. In the future, researchers should conduct randomized controlled clinical studies with a higher number of cases.
